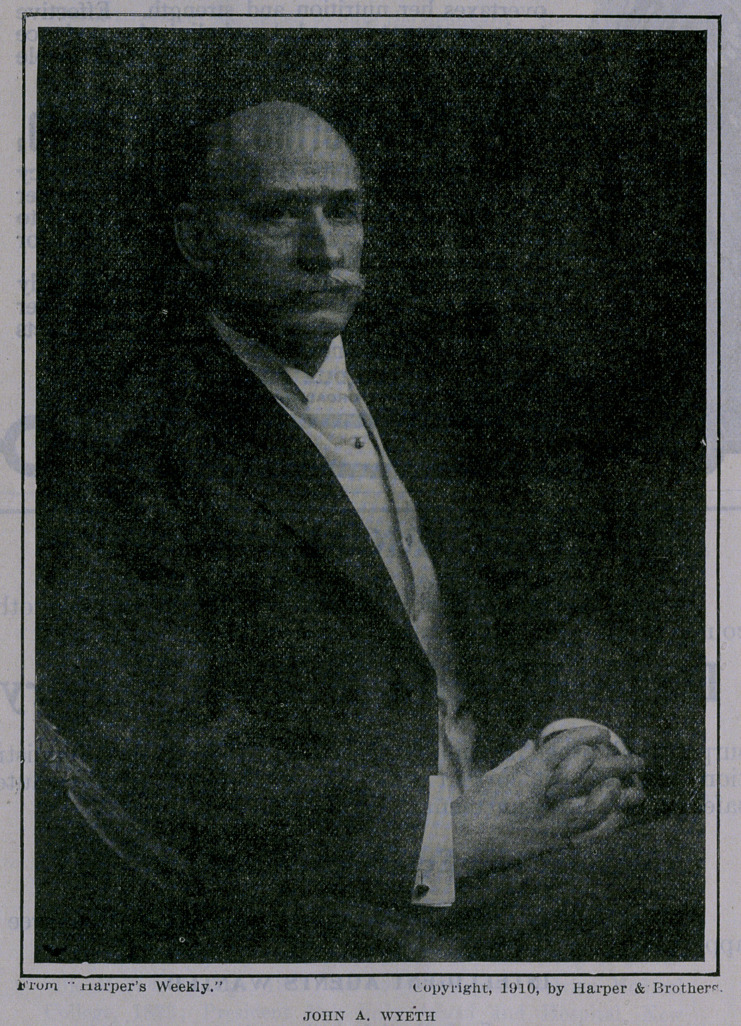# Books and Magazines

**Published:** 1911-05

**Authors:** 


### Books and Magazines.

SURGERY. By John Allan Wyeth, M. D., LL. D. (University of Alabama); President of the New York Academy of Medicine; President of the Medical Faculty of and Surgeon-in-Chief to the New York Polyclinic Medical School and Hospital; ex-President American Medical Association, of the New York State Medical Association, and of the New York Pathological Association; Honorary Member Texas State Medical Association, etc., etc. 864 illustrations, 57 in colors. Price, $3.00; postage, 60 cents. Cloth and gilt. Marion Sims Wyeth & Co., 244 Lexington Avenue, New York.

This monumental work on surgery has run through several editions. Thirty-five hundred copies of the last edition, issued by the Appletons, were sold at $6.00 per copy. This latest and thoroughly revised edition is issued by Dr. Wyeth, printed by his son's company, and is on the market at $3.60, delivered. It is dedicated to Dr. Wyeth's father-in-law, the illustrious J. Marion Sims, "whose brilliant achievements carried the fame of American surgery throughout the civilized world."

A word of commendation of this famous work would be like commending the Standard Dictionary or the United States Dispensatory. It will be the standard for the next fifty years. Dr. Wyeth is the idol of the medical profession of the South. He is an Alabamian. He rode and fought with Forrest, and wrote the biography of that immortal cavalryman.

---

John Allan Wyeth, Alabama, 1845; M. D., University of Louisville Medical College, 1869; *Ad eundem* degree, Belvieu Medical College, 1873; President Polyclinic School and Hospital, New York; ex-President A. M. A.; ex-President New York Academy of Medicine; Honorary Member Texas State Medical Association; author of numerous medical and surgical text-books and miscellaneous works, amongst them a "Life of Forrest, the Confederate Cavalry Chieftain," with whom he fought in the 60's, enlisting when he was 17 years of age; married Florence Nightingale Sims,

From "Harper's Weekly." Copyright, 1910, by Harper & Brothers.

JOHN A. WYETH

daughter of Dr. J. Marion Sims. See Book Notices for review of Dr. Wyeth's monumental work on surgery, this issue.

## Figures and Tables

**Figure f1:**